# Cross-cultural adaptation and measurement properties of the Dutch knee self efficacy scale (K-SES)

**DOI:** 10.1186/s13102-019-0115-y

**Published:** 2019-03-07

**Authors:** Wim van Lankveld, Nicky van Melick, Bas Habets, Yvette Pronk, J. Bart Staal, Robert van Cingel

**Affiliations:** 1 Research group Musculoskeletal Rehabilitation Nijmegen, HAN University of Applied Sciences, Kapittelweg, 33 Nijmegen, The Netherlands; 2 Research Institute for Health Sciences, IQ healthcare, Radboud University Medical Center, Nijmegen, The Netherlands; 3 Knee Expert Centre, Eindhoven, The Netherlands; 4 Sport Medical Centre Papendal, Arnhem, The Netherlands; 5 Rudolf Magnus Institute of Neurosciences, Department of Rehabilitation, Physical Therapy Science & Sports, University Medical Center Utrecht, Utrecht, The Netherlands; 6 Research Department, Kliniek ViaSana, Mill, The Netherlands

**Keywords:** Anterior cruciate ligament, Reconstruction, Self-efficacy

## Abstract

**Background:**

Self-efficacy is related to outcome after anterior cruciate ligament (ACL) tears. The Knee Self Efficacy Scale (K-SES) available in Swedish and English, was developed to measure self-efficacy in present (K-SES_present_) and future (K-SES_future_) functioning. The objective of this study was to determine measurement properties of the K-SES in Dutch patients.

**Methods:**

The K-SES was translated and structural validity, internal consistency, test-retest reliability, and measurement error were assessed in three patient samples: one group completed the questionnaire and additional measures pre-surgery (*N* = 200), and one group post-surgery (*N* = 58). The third group (post-surgery) completed the K-SES twice (*N* = 50).

**Results:**

Exploratory factor analysis distinguished two underlying important factors: K-SES_present_ and K-SES_future_. However, the distinction was not confirmed in Confirmatory Factor Analysis (CFA). Internal consistency for both subscales was excellent (Cronbach’s alpha > .80). Test-retest reliability absolute agreement was 0.95. A-priori formulated hypotheses on the relation between Knee Self Efficacy Scale Dutch (K-SES-D) and related constructs were confirmed. Moderate to high correlations (*r* > 0.50) were reported with Knee Injury and Osteoarthritis Outcome Score (KOOS) before reconstruction. High negative correlation was found with fear of movement and pain catastrophizing (*r* < − 0.60), and low correlation (*r* < 0.50) with locus of control and measures of distress.

**Conclusion:**

Acceptability, internal consistency and test-retest reliability of the K-SES-D subscales are satisfactory. Construct validity of both subscales was confirmed by exploratory factor analysis and hypothesis testing. However, construct validity was not confirmed in CFA. Further research is needed to test responsiveness.

**Electronic supplementary material:**

The online version of this article (10.1186/s13102-019-0115-y) contains supplementary material, which is available to authorized users.

## Background

An anterior cruciate ligament (ACL) tear is a frequently reported injury in sports activities that include pivoting movements of the knees, such as soccer, basketball, football, handball, and skiing. The incidence of ACL tears is estimated at 68.6 per 100,000 person years [1]. An ACL tear is a painful condition and has a large impact on a patient’s functioning [2]. Both conservative and reconstructive (ACLR) treatment options are available [3]. However, most athletes in pivoting sports will opt for an ACLR procedure. Rehabilitation after ACLR involves exercises and training to enhance the athlete’s performance in sports activities [4]. Despite extensive rehabilitation, it is estimated that only 50–65% of the patients with ACLR return to their pre-injury level of sports [5, 6].

Psychological factors are important predictors of return to sport in injured athletes in general [7], and this also applies to ACL injured athletes [8, 9]. Psychological variables related to return to sport in ACLR include catastrophizing cognitions and kinesophobia [10], levels of stress [11], and self-efficacy beliefs [12]. Self-efficacy beliefs also predict outcome after ACL tears [13]. A recent systematic review showed strong support for the role of self-efficacy in predicting ACLR outcome [9]. As these psychological variables are relevant for rehabilitation outcome in ACL patients, they should be assessed and addressed in ACL rehabilitation [14].

To be able to identify self-efficacy in ACL and ACLR patients, researchers have used different instruments [9]. Bandura suggested that self-efficacy should be measured as specific as possible by measuring self-efficacy beliefs related to the target behaviour [15]. In line with these recommendations, the Knee Self-Efficacy Scale (K-SES) was developed in Sweden to assess self-efficacy beliefs related to behaviour after ACL tears and is previously published in Swedish and English [16]. The K-SES consists of 22 items measuring self-efficacy in daily activities, sports and leisure activities, physical activity, and knee function in the future. Based on dimension reduction techniques, two important factors were identified [16]. The first factor refers to self-efficacy related to present functioning (K-SES_present_) measured with items related to daily activities, sports and leisure activities, and physical activities. The second factor measures knee function in the future (K-SES_future_). The K-SES has shown good internal validity, face validity, and convergent validity. In a longitudinal study, it was shown that self-efficacy assessed with the K-SES prior to surgery was a predictor of ACLR outcome 1 year after surgery [13].

However, the K-SES is not available in Dutch, and no data have been reported on the goodness of fit of the two factor model in a confirmatory factor analysis. Therefore, the aim of this study was 1) to cross-culturally adapt the K-SES into Dutch according to international accepted guidelines, and 2) to determine measurement properties of the K-SES Dutch language version (K-SES-D) including goodness of fit for the two factors.

## Methods

### Cross cultural adaptation of the K-SES

A cross cultural adaptation of the K-SES into Dutch was performed according to international accepted guidelines [17]. The K-SES items asses self-efficacy in the domains of daily activities (7 items); sports activities (5 items); physical function tasks (6 items); and knee function in the future (4 items). In a factor analysis the 22 items reflect two dimensions: self-efficacy beliefs in current functioning (K-SES_present_) and self-efficacy towards future functioning (K-SES_future_). Items are scored on an 11-point scale (0 = not certain at all and 10 = very certain). The Beaton method involves different steps of forward and backward translation using two independent translators for each step. Consensus discussions between the independent translators were used to resolve any discrepancies between translation. Acceptability of the translated Dutch version was checked in a small sample of 15 ACLR patients using structured interviews by phone. Patients were asked if they had any difficulty completing the questionnaire, and whether they had suggestions about any item.

### Measurement properties

The Consensus-based **S**tandards for the selection of health Measurement Instruments (COSMIN) [18] was used as a guide in determining measurement properties of the K-SES-D. The following COSMIN measurement properties will be described: structural validity, internal consistency, reliability, measurement error, and construct validity using hypothesis testing.

#### Patient samples

The final version of the K-SES-D was used in three different samples of ACL patients. The first sample consisted of 200 patients of Kliniek ViaSana (Mill, the Netherlands) with an ACL tear scheduled for ACLR with quadruple semitendinosus graft. All patients completed the K-SES-D on the computer, prior to reconstructive surgery, as part of a larger clinical prospective cohort study. Inclusion criteria for this study were: age between 16 and 50 years, Tegner Activity Scale ≥6, undergoing ACLR. Excluded were patients undergoing revision surgery, or patients with an ACL tear of the contralateral knee in the past.

The second sample consisted of 72 ACLR patients, not included in sample 1, who were invited to participate in a study conducted in 2015 to develop the Photographic Series of Sports Activities for Anterior Cruciate Ligament Reconstruction (PHOSA-ACLR), a new instrument to measure fear of harm or re-injury after ACLR [19]. Patients in sample 2 were included in this study between 3 and 36 months after reconstruction. In 2016, a third sample of 50 ACLR rehabilitation patients completed the K-SES-D on paper twice with an interval of 1 week. Additional file 1: Figure S1 shows details about the 3 independent samples in the study and the purpose they were used for.

Patients in sample 2 and 3 with an ACLR were invited through direct referral by two physical therapists working in Sports Medical Centres (Sports Medical Center Papendal, Arnhem, the Netherlands; and Funqtio, Steyl, the Netherlands), or through referral from primary care physical therapists in the Nijmegen area associated with the HAN University of Applied Sciences. Patients older than 16 years of age were asked to participate in this study if they had undergone ACLR. Exclusion criteria were: unable to read or write Dutch, and co-morbidity affecting knee function. Questionnaires were filled in during a visit to the physical therapist using a hardcopy query. When data were incomplete the patient was asked to complete the questionnaire.

#### Measurements

All participants reported demographics, and time since ACLR. Based on the K-SES-D scores, average item scores were computed for K-SES-D_present_ (18 items), K-SES- D_future_ (4 items), and K-SES-D_total_ (22 items). To be able to compare samples, knee related function was assessed in samples 1 and 2 using the validated Dutch version of the Knee Injury and Osteoarthritis Outcome Score (KOOS) [20]. The KOOS measures pain, other disease-specific symptoms, activities of daily life (ADL) function, sport and recreation function, and knee-related quality of life (QOL). For each scale the scores were recoded from 0 to 100, with 100 depicting no problems.

To determine structural validity of the K-SES-D, sample 2 completed a set of additional questionnaires. Fear of movement was assessed using the Dutch version of the Tampa Scale of Kinesiophobia (TSK) [21]. Catastrophizing cognitions regarding pain were measured using the validated Dutch version of the Pain Catastrophizing Scale (PCS) [22]. Health Locus of Control was assessed using the validated Dutch version of the Multidimensional Health Locus of Control Scale (MHLC) [23], measuring internal, external, and physician locus of control. Finally, distress was measured using the validated Dutch version of the Hospital Anxiety and Depression Scale (HADS) [24]. For each scale in the study the validated Dutch version was used, and a higher scores means that the patients reports higher levels of the assessed construct.

Data of sample 1 and 2 were sampled using a web based program, and patients had to complete every question. Therefore, there are no missing individual items in these samples. Sample 3 completed a paper version of the questionnaire, and data completeness was checked when patients handed in the questionnaires. When data were incomplete the patient was asked to complete the questionnaire.

#### Data analysis

Metric data were described using means, and standard deviations (SDs). Item descriptives and reliability were studied in sample 1 and 2 in a similar way as described by Thomeé et al. [16]. Measures of Skewness between − 2 and + 2 are considered acceptable for normal univariate distribution. The strength of pearson correlations between variables is defined as negligible (.00 to .30), low (.30 to .50), moderate (.50 to .70), high (.70 to .90), and very high (.90 to 1.00, [25]). Differences between independent groups were tested using the T statistic, and significance of the difference is reported. Regression analysis was used to determine the multiple correlation (R) between a set of independent variables and the subscales of the K-SES-D. For these multiple regressions, both multiple correlation (R), and percentage of variation explained (R^2^) were reported.

*Structural validity* was investigated using Principal Component Analysis (PCA). As the structure of the original K-SES reflects two important factors of self-efficacy. The first factor refers to self-efficacy related to present functioning (K-SES_present_) with items related to daily activities, sports and leisure activities, and physical activities. The second factor measures self-efficacy beliefs related to knee function in the future (K-SES_future_). [16], it is expected that the same two important factors will be observed in this study. Therefore, the underlying factor structure of the K-SES-D in combined data from sample 1 and 2 was analysed using PCA [26]. First, sampling adequacy was determined using Kaiser-Meyer-Olkin (KMO) and Bartlet test. Sampling adequacy is considered good when KMO value >.8, and Bartlett test of sphericity is significant. Next, a maximum likelihood PCA with Harris Kaiser’s rotation in a two factor solution was conducted. It was expected that the items related to present physical performance would have significant loadings on the first factor, and that the items of the scale knee function in the future would have significant loadings on the second factor. In addition, a Confirmatory Factor Analysis (CFA) was used to test whether the observed item scores of the K-SES-D in sample 1 and 2 fit the two factor model. In CFA different indicators are used to depict goodness of fit [27]. In this study four indices of absolute fit were used: Chi-squared test corrected for degrees of freedom (CMIN/DF), the Root Mean square error of estimation (RMSEA), the Comparative fit index (CFI), and the standardised root mean square residual (SRMR). Acceptable fit is indicated by CMIN/DFD < 5.0, RMSA < 0.6, CFI > 0.90, and SRMR < 0.08 [27]. *Three measurement properties of reliability* were assessed for this study: internal consistency, test-retest reliability, and measurement error [18]. Internal consistency was calculated using Cronbach Alpha. A value of Cronbach Alpha >.80 is considered excellent [28]. Cronbach alpha was calculated separately for K-SES-D_present_, K-SES-D_future_, and K-SES-D_total_ in samples 1 and 2. Test-retest reliability was calculated based on two independent assessments of the instrument in sample 3. Mean differences between test and re-test item scores were calculated with corresponding 95% confidence interval (CI). When zero lies within the 95% CI this is considered a criterion for absolute agreement. Finally, for each K-SES-D scale intraclass correlation coefficients (ICC) between both assessments were calculated with corresponding 95% CI to determine absolute agreement between assessments. The two way random effects model was used. An ICC above 0.75 is considered good [29]*.* Standard error of measurement (SEM) was computed in the same sample by dividing the SD of the mean difference between both assessments (SDdiff) by √2 [29].

*Construct validity* was further investigated by determining the degree to which the scores on the instrument are consistent with a-priori defined hypotheses. Table 1 shows the hypotheses that have been formulated based on the reported construct validity of the original manuscript. It was expected that exploratory factor analysis would result in 2 important factors: K-SES-D_present_ and K-SES-D_future_. K-SES-D_present_ was expected to be highly correlated with self-reported knee functioning, whereas lower correlations were expected for K-SES_future_. Furthermore, it was expected that KSES_present_, and K-SES_future_, would have moderate to high reversed correlations with levels of both fear of movement, and catastrophizing, and low to moderate correlations with locus of control and distress. The construct validity is good when > 75% of the a-priori formulated hypotheses about the relation of the construct with other theoretically derived constructs are confirmed [29].

**Table 1:** Hypotheses about the relation between K-SES-D and other variables

1	K-SES-D will show 2 dimensions in exploratory factor analysis: present and future self-efficacy.
2	K-SES-D will fit the proposed 2 dimension model using confirmatory factor analysis.
2	K-SES-D_present_ will have a high positive correlation with knee function (KOOS) (0.50 < r < 0.80).
3	K-SES-D_future_ will have a low correlation with knee function (KOOS) (0.30 < r < 0.50).
4–5	K-SES-D_present_ and K-SES-D_future._, both have moderate to high correlations with TSK (0.50 < r < 0.80).
6–7	K-SES-D_present_, K-SES-D_future._ both will have strong correlations with PCS (0.50 < r < 0.80).
8	K-SES-D_present_, K-SES-D_future.,_ both will have low association with MHLC (0.30 < r < 0.50).
9	K-SES-D_present_, K-SES-D_future._, both will have low association with HADS (0.30 < r < 0.50).

Abbrevations: *K-SES-D*_*present*_ Knee Self-Efficacy Scale present function, *K-SES-D*_*future*_ Knee Self-Efficacy Scale present function, *KOOS* Knee Injury and Osteoarthritis Outcome Score, *TSK* Tampa Scale of Kinesiophobia, *PCS* Pain Catastophizing Scale, *MHLC* Multidimensional Health Locus of Control, *HADS* Hospital Depression and Anxiety Scale

## Results

Consensus was reached in the translation panel with only minor differences resolved. The final Dutch version was endorsed by 15 patients receiving physical therapy treatment after an ACLR (9 males (60%), mean age is 21 years (S.D. = 2.8), average number of months since reconstruction is 4 (S.D. = 5.6*).* The back-translation was endorsed by the author of the original K-SES study (personal communication R. van Cingel).

Table 2 displays the demographic characteristics of both sample 1 and 2 and the scores for self-reported knee function scales.

**Table 2:** Characteristics of patients in the ACL tear group pre-surgery (sample 1: ACL) and in rehabilitation after reconstruction surgery (sample 2: ACLR), and T value for independent samples

	ACL (*N* = 200)	ACLR (*N* = 58)	T.
Age (mean; SD)	23.7 (8.5)	25.9 (8.2)	n.s.
Gender: % male	69.0%	43.0%	
KOOS pain (0–100) (mean; SD)	70.0 (18.2)	81.8 (17.7)	4.3**
KOOS symptoms (0–100) (mean; SD)	63.5 (20.7)	63.2 (12.8)	n.s.
KOOS ADL (0–100) (mean; SD)	82.0 (17.7)	89.1 (14.2)	2.8**
KOOS Sports and Leisure (0–100) (mean; SD)	37.7 (26.6)	59.1 (30.6)	5.2**
KOOS QOL (0–100) (mean; SD)	38.7 (16.7)	48.3 (14.1)	3.9**

Abbrevations: *ACL* Anterior Cruciate Ligament, *ACLR* Anterior Cruciate Ligament Reconstruction, *N* number of participants, *n.s.* not significant, *KOOS* Knee Injury and Osteoarthritis Outcome Score, *ADL* Activities of Daily Life, *QOL* Quality of Life

**p < .001

Average number of months since ACL for patient on the waiting list in sample 1 was 6.7 months (S.D. = 11.1). In sample 2, 58 (77%) of the 75 eligible patients participated. Time since reconstruction is 12.9 month (range = 2–48). The proportion of males was higher in the ACL group awaiting surgery (Z score for difference between two independent proportions Z = 3.6, *p* < .001). The ACL group awaiting surgery reported poorer scores in KOOS pain, ADL, Sports and Leisure, and QOL (*p* < .001) when compared to the ACLR group (sample 2). The difference in KOOS subscale scores may be expected as the average time since ACL between both groups is very different. Item characteristics of the K-SES-D items of sample 1 and 2 were analysed first. Item scores ranged from 0 to 10 for each item. All items showed symmetry of frequency distribution, with measured of Skewness for all items < |1|.

### Structural validity

Factor analysis in the combined data from sample 1 and 2 was adequate (KMO = 0.95, and Bartlet test significant < .001). PCA using Harris Kaiser’s factor rotation resulted in 2 important factors. Items of the K-SES-D reflecting present physical performance/function had high loading on the first factor (all factor loadings > 0.70). This factor K-SES-D_present_ explained 56% of the variance of the K-SES-D total. The second factor (K-SES-D_future_) explained an additional 12% of the variation in item scores with only the four items of the knee function in the future subscale demonstrating high loadings. In a confirmatory factor analysis the two factor model could not be confirmed. Only two of the four indices indicated acceptable fit of the model (SRMR = 0.05; CMIN/DF = 4.9; CFI = 0.85; RMSEA = 0.12). Inspection of the covariance matrix showed that in particular the 18 items relating to K-SES-D_present_ did not fit well into the CFA.

### Reliability

Average scale scores were calculated for K-SES-D_present_ and K-SES-D_future_ and K-SES-D_total_ score, both in sample 1 (ACL), and 2 (ACLR). In Table 3 scale statistics are given for both subscales of the K-SES and total score for both samples.

**Table 3:** Descriptive data on final K-SES-D from two independent samples: sample 1 (ACL patients; *N* = 200), and sample 2 (ACLR patients; *N* = 58)

	K-SES-D_present_		K-SES-D_future_		K-SES-D_total_	
	ACL	ACLR	ACL	ACLR	ACL	ACLR
Mean	4.6	7.9	6.2	5.3	5.4	7.2
SD	2.3	2.0	2.1	2.6	1.7	1.9
Cronbach’s Alpha	0.92	0.91	0.83	0.81	0.95	0.96
95% CI lower	4.3	7.4	5.9	4.6	4.7	6.7
95% CI upper	4.9	8.4	6.5	6.0	5.3	7.7
Observed range	0–10	0–10	0–10	0–10	0–10	0–10
Skewness	0.15	−1.1	−0.56	− 0.25	0.12	− 0.98

Abbrevations: *K-SES-D* Knee Self Efficacy Scales Dutch, *ACL* Anterior Cruciate Ligament, *ACLR* Anterior Cruciate Ligament Reconstruction, *SD* Standard Deviation, *CI* confidence interval

Cronbach’s Alpha as a measure of internal consistency for each subscale is good, and measures of skewness indicate normal distribution. K-SES-D_present_ in the ACL group was significantly lower compared to mean score in the ACLR group (T = 9.9, *p* < .0001). With regard to K-SES-D_future_ the relation was reversed: ACL patients reported higher levels of efficacy beliefs with regards to their future knee functioning compared to ACLR patients (T = 2.71, df = 256; *p* < .001).

Reliability or test-retest reliability for the K-SES-D_present_ and K-SES-D_future_ subscales was computed in sample 3 consisting of 50 ACLR patients. Average age in this sample was 25.8 years (range 16–51), with 57% males, and time since reconstruction was on average 6 months (range 2–21). Average change score for K-SES-D_present_ was − 0.36 (95% CI -0.62,-0.09), with SD = 0.92. Test-retest ICC between both assessments was 0.93 (95% CI 0.88,0.96) and SEM was 0.13. Average change score for K-SES-D_future_ was − 0.14 (95% CI -0.35,0.08), with SD = 0.74. Test-retest ICC between both assessments was 0.92 (95% CI 0.86,0.95) and SEM was 0.46.

### Construct validity (using hypothesis)

Correlations were computed between measures of self-efficacy on the one hand, and knee function variables in sample 1 and 2 on the other hand. Table 4 shows the correlations between K-SES-D subscales on the one hand, and self-reported knee functioning assessed with the KOOS subscales on the other. For both K-SES subscales multiple correlation (R), and percentage of variation explained (R^2^) are given depicting the strength of the relation between the combined set of KOOS scales with K-SES-D scales.

**Table 4:** Pearson correlation (95% Confidence interval) between K-SES-D and KOOS self-report knee function scales in sample 1 (ACL) and 2 (ACLR)

	K-SES-D_present_		K-SES-D_future_		K-SES-D_total_	
KOOS	ACL	ACLR	ACL	ACLR	ACL	ACLR
Pain	.64(.53,.73)	.82(.89,.67)	.16(.00,.31)	.54(.33,.72)	.61(.48,.72)	.82(.68,.89)
Sympt	.44(.30,.55)	.27(.05,.59)	.15(.00,.32)	.37(.10,.59)	.43 (31,.56)	.34(.00,.64)
ADL	.63(.51,.72)	.82(.64,.90)	.16 (.01,.33)	.46(.24,.67)	.60(.47,.71)	.79(.61,.69)
Sport	.70(.59,.79)	.89(.67,.89)	.13(.00,.28)	.60(.41,.76)	.66(.53,.76)	.82(.70,.90)
Qol	.64(.41,.38)	.59(.35,.78)	.32(.18,.46)	.39(.13,.63)	.57(.44,.67)	.59(.37,.77)
R	.75**	.85**	.33**	.66**	.73**	.87**
R^2^	.57**	.74**	.11**	.42**	.54**	.75**

Abbrevations: *K-SES* Knee-Self Efficacy Scale, *ACL* Anterior Cruciate Ligament *ACLR* Anterior Cruciate Ligament Reconstruction, *KOOS* Knee Injury and Osteoarthritis Outcome Score, *Pain* KOOS pain, *Sympt* KOOS symptoms, *ADL* KOOS Activities of Daily Activities, *Sport* KOOS Sports and Leisure Activities, *QOL* KOOS quality of Life, *R* multiple correlations; *R*^*2*^ percentage of variation explained

**p < .001

With the exception of the KOOS symptom subscale, all correlations between KOOS subscales and K-SES-D_present_ scores were moderate to high.

In Table 5, correlations are given between K-SES-D_present_, K-SES-D_future_, and K-SES-D_total_ scores on the one hand, and the psychological variables on the other hand in sample 1. Psychological variables include fear of movement, pain catastrophizing, health locus of control, and anxiety and depression.

**Table 5:** Correlation (95% Confidence Interval) between K-SES-D subscales and self-report psychological variables

	K-SES-D_present_	K-SES-D_future_	K-SES-D_total_
TSK	−0.66 (−0.51, −0.78)	−0.65 (− 0.46, − 0.78)	−0.74 (− 0.61, − 0.83)
PCS	−0.64 (− 0.40, − 0.79)	−0.54 (− 0.34, − 0.72)	−0.67 (− 0.42, − 0.80)
MHLC Intern	0.10 (− 0.16, 0.40)	−0.35 (− 0.11, − 0.57)	−0.19 (− 0.19, − 0.48)
MHLC Extern	0.32 (0.09, 0.57)	0.34 (0.11, 0.55)	0.36 (0.10, 0.59)
MHLC Physician	0.15 (− 0.12, 0.44)	0.28 (0.04, 0.52)	0.20 (0.10, 0.49)
HADS	− 0.29 (− 0.06, − 0.56)	−0.34 (− 0.04, − 0.56)	−0.32 (− 0.05, − 0.37)
R	0.74**	0.71**	0.80**
R^2^	0.55**	0.51**	0.63**

Abbrevations: *K-SES-D* Knee Self Efficacy Dutch, *TSK* Tampa Scale Kinesophobia, *PCS* Pain Catastrophizing Scale, *MHLC* Multidimensional Health Locus of Control Scale, *HADS* Hospital Anxiety and Depression Scale, *R* multiple correlations, *R*^*2*^ percentage of variation explained

**p < .001

K-SES-D scale scores had moderate to high (*r* > |.50|) negative correlations with TSK, and PCS. Negligible to low correlations were found between with MHLC scale scores, and HADS score. Together, these variables explained 55, 51, and 63% of the variation in the K-SES scales. Three stepwise regression analyses were performed with K-SES-D_present_, K-SES-D_future_, and K-SES-D_total_ scores as dependent variables. Self-reported knee function assessed using the KOOS subscales explained 74% of the variance in K-SES-D_present_. Entering the combined psychological variables explained an additional 7% of the variation in K-SES-D_present_ (F change = 2.4; df = 6,46; *p* < .05), 21% of variation K-SES-D_future_, and 7% of the variation in K-SES-D_total_ (F change = 3.2; df = 5, 47; *p* < .05).

All except one of the hypotheses formulated in Table 1 were confirmed in the analysis. Hypotheses 2 was rejected as the FCA did not confirm a 2 factor model underlying the data.

## Discussion

Measurement properties of the translated K-SES-D were good, with good reliability and validity. Exploratory factor analysis revealed two factors underlying the scores on the K-SES-D similar to the factors reported in the original study: self-efficacy beliefs about daily activities, sports and leisure activities, and physical activity (K-SES-D_present_), and self-efficacy beliefs about knee function in the future (K-SES-D_future_). However, in a CFA, this hypothesised 2-factoral model could not be confirmed. More than 75% of a-priori formulated hypotheses were confirmed.

The negligible to low correlation with Internal Health Locus of Control underlines the importance of measuring self-efficacy beliefs as specific as possible related to the target behaviour [15]. As rehabilitation after ACLR involves exercises and training to enhance the patient’s performance in sports activities [30], there is a need to understand the athlete’s beliefs regarding exercise and training. The K-SES assesses these beliefs [16], but item responses did not fit the hypothesised two factor model in a CFA. This means that the items of this scale are not consistent with a researcher’s understanding of the nature of that construct (or factor) [29]. In particular, the items on the K-SES-D_present_ factor do not fit well in the model. These findings are consistent with the way the instrument was developed. The authors generated items to be categorized in four a-priori defined groups: daily activities, sports and leisure activities, physical activities, and knee functioning in the future [16]. In an exploratory factor analysis of the original data, which was replicated in this study, two important factors emerged, K-SES_present_, and K-SES_future_. Items developed to measure self-efficacy related to present daily activities, sports and leisure activities, and physical activities, are combined in the composite K-SES_present_ scale. The meaning of the average K-SES_present_ score is therefore not easy to interpret. As might be expected, K-SES-D_present_ is strongly correlated with self-reported functioning of the knee assessed with the KOOS, showing large overlaps in variation. The 4 items reflecting K-SES-D_future_ did not pose any problem in the CFA, and the scale score showed low correlations with current knee function. In addition, K-SES_future_ has been shown to be a better predictor of future functioning compared to K-SES_present_ [13]. However, as ACLR rehabilitation involves extensive exercises and training [4], the patients beliefs with regard to training and exercise are also important to the physical therapist.

This study is not without limitations. The selection of patients makes it hard to make direct comparisons between this study and outcomes reported in the original Swedish K-SES validation study. In the original study, ACL and ACLR patients were both used to determine different measurement properties [16]. However, although the samples of patients included in this study differ from the samples in the original study, average item scores in both factor obtained in the current study are similar to item scores reported earlier [13]. Furthermore, patients were not assessed at a fixed moment in time after ACL (sample 1) or ACLR (sample 2). It is unclear whether the reported differences in time since ACL or ACLR will impact these results. Another drawback of this study is that there are no data available on responsiveness of the K-SES-D. The original K-SES was shown to be sensitive to change as scores increased during rehabilitation after ACL tear [31]. Further research is needed to determine to what extent the K-SES-D is responsive to change. Finally, in this study data for test-retest reliability were assessed with an interval of 1 week. This interval was chosen to accommodate patients and therapists, and avoid additional visits to the physical therapist. However, COSMIN recommends an interval of 2 weeks to avoid recall of the items. Therefore, we do not know whether recall of items poses a problem in this study.

Despite these limitations, the study has important findings. The K-SES-D measures self-efficacy beliefs related to functioning in ACL and ACLR patients. Acceptability, internal consistency, and test-retest reliability of the subscales are good. Construct validity was confirmed in principal component analysis and hypothesis testing, but not in a confirmatory factor analysis. These self-efficacy beliefs are only weakly correlated to general self-efficacy beliefs. In ACLR patients, self-efficacy beliefs towards present functioning (in particular towards physical activity exercise) are different from self-efficacy beliefs about future functioning. Addressing these self-efficacy beliefs is important as most patients are motivated to return to previous levels of sports [32], and self-efficacy predicts future function [13]. Furthermore, self-efficacy is recognized as one of the main psychological factors associated with return to sports [33], and satisfaction with knee function after an ACLR [34]. A number of different available techniques to improve self-efficacy beliefs have been studied [35]. However, only one study reported on the effects of an intervention addressing self-efficacy in ACL patients [36]. This study concluded that a strategy to improve self-efficacy beliefs as part of a standard rehabilitation protocol did not result in a better outcome compared to a standard protocol. However, the study was limited in size, and no inclusion criteria related to self-efficacy were used. Interventions are likely to be more effective when addressing only those patients with low self-efficacy. In recent years, studies in ACL and ACLR have concluded that an individual, or tailored, approach is needed to increase return to sports [37]. Including only patients with low K-SES_future_ in self-efficacy targeting interventions is likely to increase self-efficacy in these patients. Furthermore, other modes of interventions should be addressed as well. Cognitive behavioural approaches as described in the study of Thomeé [36] heavily depends on social persuasion as a way to improve self-efficacy. Interventions using an exposure based approach within the fear-avoidance model should be considered as well [38]. Such interventions might also hold a stronger appeal for patients.

## Conclusions

The translated Dutch version of the K-SES was acceptable for patients. Internal consistency and test-retest reliability of the K-SES-D subscales are satisfactory. The subscales measure self-efficacy related to present functioning (K-SES_present_), and self-efficacy beliefs related to knee function in the future (K-SES_future_). Construct validity of both subscales was confirmed by factor analysis and hypothesis testing. However, construct validity was not confirmed using a two model solution in CFA. Further research is needed to test responsiveness.

## Additional file

Additional file 1: **Figure S1.** Samples used in the study. (DOCX 34 kb)

## Abbreviations

ACL: Anterior Cruciate Ligament
ACLR: Anterior Cruciate Ligament Reconstruction
ADL: Activities of Daily Life
CFA: Confirmatory Factor Analysis
CFI: Comparative Fit Index
CMIN/DF: Chi-square corrected for Degrees of Freedom
COSMIN: Consensus based standard for the selection of health measurement instruments
FoH: Fear of Harm
HADS: Hospital Anxiety and Depression Scale
ICC: Intraclass Correlation Coefficient
KMO: Kaiser-Mayer-Olkin test
KOOS: Knee injury and Osteoarthritis Outcome Scale
K-SES: KNEE Self-Efficacy Scale
K-SES_future_: Knee self-efficacy related to future functioning
K-SES_present_: Knee self-efficacy related to present functioning
MHLC: Multidimensional Health Locus of Control
PCA: Principal Component Analysis
QOL: Quality of life
RMSE: Root Mean Square Error of Estimation
SEM: Standard Error of Measurement
SRMR: Standardized Root Mean square Residual
TSK: Tampa Scale of Kinesophobia
