# Supplementary material for: Cross-cultural adaptation and measurement properties of the Dutch knee self efficacy scale (K-SES)
Source: BMC Sports Sci Med Rehabil. 2019 Mar 7;11:3. doi: 10.1186/s13102-019-0115-y (PMC6407177; doi:10.1186/s13102-019-0115-y)
Supplement: Supplementary file 1 [file 13102_2019_115_MOESM1_ESM.docx]

Sample 1	Sample 2	Sample 3
Patients with ACL waiting list reconstruction.<br><br>N = 200<br><br>Used to determine internal consistency and construct validity	Patients in ACL Reconstuction rehabilitation.<br><br>N = 72<br><br>Used to determine internal consistency and construct validity	Patients in ACL Reconstruction rehabilitation.<br><br>N = 50<br><br>Used to determine test-retest reliability
